# Reply to: [Letter to the Editor] 

**DOI:** 10.1089/heq.2024.0049

**Published:** 2024-09-12

**Authors:** Justin M. List, David Au, William C. Yarbrough, Ernest Moy

**Affiliations:** ^1^ VA Office of Health Equity, Washington, District of Columbia, USA.; ^2^ Health Services Research and Development, VA Puget Sound Health Care System, Seattle, Washington, USA.; ^3^ The University of Texas Southwestern Medical Center, Dallas, Texas, USA.; ^4^ VA North Texas Health Care System, Dallas, Texas, USA.

To the Editor:

Members of the Jesse Brown for Black Lives Task Force Clinical Algorithms Committee at the Jesse Brown Veterans Affairs Medical Center have raised concern about the process for Veterans Health Administration (VHA) adoption of the of the Global Lung Function Initiative (GLI) average reference equation (GLI Global) in the interpretation of pulmonary function testing (PFT). List et al. provide four examples of VHA efforts to eliminate or mitigate the use of race in clinical decision support tools.^1^ In contrast to the 2021 Chronic Kidney Disease Epidemiology Collaboration (2021 CKD-EPI) equation example, the authors noted significant differences in the approach and underlying data that led the American Thoracic Society (ATS) recommendations to adopt use of a single reference equation.

As stated in our original article, we agree with the letter to the editor (LTE) authors that race is a social construct and recognize the important work by Lundy Braun reporting on the historical record of lung measurement to justify enslavement and racial inferiority.^2^ Although we share the core values stated in the LTE, VHA remains concerned about an uninformed and unstructured implementation process that has the potential to create other inequities and roll back achievements in health outcome equity. One of the VHA’s missions is the provision of equitable delivery of health care services, and it has an obligation to understand the implication of changes in that delivery on health care outcomes.

The LTE states “Based on an exhaustive review of the evidence…,” the ATS reached the conclusion to transition to the universal use of GLI Global. While we embrace the studies cited by the ATS and the LTE’s authors that demonstrate better alignment *in symptoms* among Black patients, it is not clear that the evidence base has comprehensively considered the potential impact of this change *on clinical outcomes*. The literature is largely absent in important areas including lacking effects on outcomes for many clinical conditions and among diverse populations. The ATS statement posited expected effect of changes on employment and care delivery but lacked empirical evidence for overall net benefit on outcomes. In contrast to effects on clinical care delivery, such as a clinical practice guideline, the document was void of evidence synthesis on outcomes nor the typical accompanying evaluation of the evidence.

VHA has successfully eliminated differences in treatment and lung cancer specific mortality.^3^ We were concerned with internal data that demonstrated a potential shift in predicted values may result in Black and Asian veterans being considered less often for thoracic surgery for lung cancer. Given that resection is so closely tied to superior survival outcomes compared with lung conserving treatments, we found alarming the results of Bonner et al. that demonstrated potentially marked reductions in referral for Black patients to definitive therapies when using GLI Global.^4^ Moreover, depending on the condition under consideration the same person could experience benefits and harms with this transition. For example, a Black patient could experience higher monthly compensation for disability and have a lower five-year cancer free survival. These are complex issues, require clear ability to communicate tradeoffs with patients, and deserve more than a consensus statement.

At the heart of this discourse around implementation may reflect the lack of adequate diversity underlying the cohort for GLI with points and counterpoints raised in the literature.^5,6^ In comparison to the race-free 2021 CKD-EPI equation and recently announced PREVENT (Predicting Risk of cardiovascular disease EVENTs)^7^ calculator, the GLI has a limitation the other two do not: they did not require weighting of cohorts grouped by “race,” because they are considered adequately diverse, whereas GLI is not.

In this way, the underlying equations for both are more accurately “race-free” while GLI is “race-composite.” This reality is not only an academic point, but one that potentially has clinical implications in immediately applying a global reference equation. The LTE authors and ATS statement cite four studies that show that race-neutral reference equations better predict: (1) incident chronic pulmonary disease (COPD) and mortality in population-based groups;^8,9^ (2) functional capacity, symptoms, and emphysema in individuals with COPD;^10^ and (3) improved matching in the prevalence of emphysema in specific ranges of percent predicted forced expiratory volume at one second between Black and White individuals.^11^ While these are encouraging for making a switch, we do not think there exists sufficient understanding of the impact across disease conditions and racial/ethnic groups. Because the recommendations fall across racial lines, inducement of changes to benefit and health care delivery have both potential legal and constitutional issues around equal protections.

As a result, the National Pulmonary and Critical Care Program has taken a deliberative approach. The office has collaborated across the organization including with Center of Care and Payment Innovation, National Surgery Office, Veteran Engagement Office, Veteran Benefits Administration, Office of Health Equity, Quality Enhancement Research Initiative, and Health Services Research & Development researchers. The VA National Center for Ethics evaluated the evidence and concluded and agreed with the consensus that the use of race had “insufficient ethical justification…. However, deciding whether and how VHA should implement a race-neutral equation for PFT interpretation requires considering VHA’s ethical obligations both to identify and reduce racial health disparities as well as to eliminate structural racism in care. Because there is considerable uncertainty about the consequences of using a race-neutral equation, implementing such an approach will require a broader understanding of the specific benefits and harms, their magnitude, in which populations, and if they create, reduce, or worsen health disparities. …any changes to its standards using open, transparent communication to maintain and build trust with the Veterans it serves.”^12^

Earlier this year, VHA issued a letter to the ATS expressing concerns with the universal use of GLI Global. Approximately 30% of VHA facilities have transitioned to GLI Global. We have asked the field to pause further rollout, allowing VHA to understand the effects of this transition on care delivery and outcomes.

While we understand the lens from which the letter authors make this claim, we disagree with their perspective that this deliberative approach “ignores the fact that race is not a biological determinant of pulmonary function.” The work outlined above is not set up to defend a race-specific approach; rather, using quality and patient safety principles, it aims to guide the care of veterans with a race-neutral approach that ATS prematurely left to the field to determine. Finally, while disagreement on the evaluation and implementation strategy of the ATS’ recommendations may not be resolved, we continue to engage with facilities who both agree and disagree with this approach and appreciate the opportunities for continued dialogue.

## Abbreviations Used

2021 CKD-EPI: 2021 Chronic Kidney Disease Epidemiology Collaboration
ATS: American Thoracic Society
COPD: chronic pulmonary obstructive disease
GLI: Global Lung Initiative
LTE: Letter to the Editor
PFT: pulmonary function testing
PREVENT: Predicting Risk of cardiovascular disease EVENTs
VHA: Veterans Health Administration
